# The Use of New Digital Information and Communication Technologies in Psychological Counseling during the COVID-19 Pandemic

**DOI:** 10.3390/ijerph17207663

**Published:** 2020-10-21

**Authors:** Artemisa R. Dores, Andreia Geraldo, Irene P. Carvalho, Fernando Barbosa

**Affiliations:** 1 Center for Rehabilitation Research, School of Health, Polytechnic Institute of Porto, 4200-072 Porto, Portugal; 2 Laboratory of Neuropsychophysiology, Faculty of Psychology and Education Sciences of University of Porto, 4200-135 Porto, Portugal; g.andreia9@gmail.com (A.G.); fbarbosa@fpce.up.pt (F.B.); 3 CINTESIS and Department of Clinical Neurosciences and Mental Health, School of Medicine, University of Porto (FMUP), 4200-319 Porto, Portugal; irenec@med.up.pt

**Keywords:** digital information and communication technologies, psychological counseling, therapy, COVID-19, coronavirus SARS-CoV-2, digital literacy

## Abstract

The use of digital information and communication technologies (ICTs) has enabled many professionals to continue to provide their services during the COVID-19 pandemic. However, little is known about the adoption of ICTs by psychologists and the impact of such technologies on their practice. This study aimed to explore psychologists’ practices related with the use of ICTs before and during the COVID-19 lockdown, to identify the main changes that the pandemic has brought and the impact that such changes have had on their practice with clients, and also identify the factors that potentially have affected such changes. The Portuguese Psychologists Association announced the study, and 108 psychologists responded to an online survey during the mandatory lockdown. The results showed that these professionals continued to provide their services due to having adopted ICTs. Comparing with face-to-face interventions, psychologists recognized that additional precautions/knowledge were needed to use such technologies. Despite the challenges identified, they described the experience with the use of ICTs as positive, meeting clients’ adherence, and yielding positive results. Psychologists with the most years of professional experience maintained their services the most, but those with average experience showed the most favorable attitudes toward the use of technologies and web-based interventions.

## 1. Introduction

The coronavirus disease (COVID-19) pandemic represents an unprecedented global challenge in our era, strongly affecting people’s lives, namely, the exercise of various professional activities [1,2]. This unique circumstance, associated with the current availability of several digital tools, has contributed exponentially to the digital revolution that we have witnessed in recent years, with impact on the social, economic, and professional domains of life [3].

In this scenario, e-Health has emerged as one viable solution to allow the continuity of the provision of health services, particularly considering the public health measures that have been taken as a result of the National Emergency State, which has limited people’s access to in-person services [4]. E-Health is broadly defined as the provision of services related to health supported by a safe and cost-effective use of information and communications technologies (ICTs) [5].

The path toward the progressive adoption of ICTs in the field of psychology had already begun before the COVID-19 pandemic, albeit in varied degrees across different countries. If documentation guiding and/or regulating professional practice is already available in some countries, a legal or normative void still exists in others. This has implications regarding the availability of the psychological services offered, which are still quite scarce in some countries, as is the case in Portugal. For example, before the COVID-19 pandemic, few Portuguese psychologists adopted guided and unguided psychological internet interventions (1.3% and 1.5%, respectively) [6], despite the already recognized advantages of this type of intervention [7,8,9,10,11].

Many of the guidelines for the online practice of psychology are the result of the work of several national and international associations and bodies (e.g., the American Psychological Association and European Federation of Psychologists’ Associations), in an effort to set forth a consensus about the practices that they aim to guide. In Portugal, after a first approval of at-distance interventions by the Ordem dos Psicólogos Portugueses (OPP; transl.: Portuguese Psychologists Association) in 2015, the OPP issued a document with the guidelines for these types of services very recently, precisely at the peak of the COVID-19 pandemic [12,13].

In its first issue, this association claimed that psychological intervention should always be conducted within the same obligations and responsibilities (i.e., ethical principles and deontological and legal norms), regardless of the format of the intervention, as defined in the Code of Ethics. Although the OPP recognized the potential benefits of web-based interventions and use of ITCs, it also launched warnings about the need for a better understanding of the effects of the different modalities of remote intervention (e.g., written, audio or audiovisual support) compared to face-to-face intervention. The OPP additionally warned of the fact that the specificities of cyber space could elude the means of control available to psychologists, which could put privacy and confidentiality at risk [12]. In its second document, entitled “OPP guidelines for professional practice: Provision of psychology services mediated by ICTs” [13], a set of recommendations for the adoption of these technological and digital resources were presented.

In addition to the OPP, other projects have been carried out in Portugal (and elsewhere) with the aim to issue good practices to be adopted for the use of these digital and technological means in the area of health in general, and of psychology in particular. As an example, the European project THERAPY 2.0—Counseling and Therapeutic Interactions with Digital Natives, financed by the ERASMUS + program, sought to issue the appropriate integration of ICTs in counseling and therapy, especially for younger populations and refugees [14,15].

In several countries, an increasing number of studies have also sought to characterize the attitudes of psychologists toward the inclusion of ICTs in their professional practice and to gather evidence about the efficacy and effectiveness of psychological interventions mediated by ICTs (e.g., [7,16,17,18,19,20,21]). Regarding the psychologists’ attitudes, the results of the studies are not consistent (e.g., [6,22]). In Portugal, a recent study assessing psychologists’ attitudes revealed a slightly negative/neutral position regarding internet interventions and greater acceptability of blended treatment interventions when compared to standalone internet interventions. However, these attitudes seem to depend on different factors, such as knowledge and training [6]. A study in other countries revealed that, among those who have employed any online means of practicing counseling and therapy, 52.97% had a positive or very positive opinion about the use of these tools. In this study, e-mail was the most widely used online tool, and the computer and smart phone were the most frequently used equipment [14]. Different circumstances and factors seem to contribute to explain the different attitudes and the adoption (or not) of this type of resources [23]. Such factors include the therapist’s theoretical model/orientation, geographic area, previous experience with the use of these resources, presence or absence of previous training, perception about the usefulness of these tools, ease with their use, and years of clinical practice [24,25,26,27,28,29,30,31].

Regarding the effectiveness of these online means, in general, studies allow us to conclude that internet interventions may be efficacious and cost-effective [6,16,17,19,20,32,33,34,35]. They seemed to be at least as effective as face-to-face interventions in a large group of clients receiving treatment for psychological disorders, namely, for generalized anxiety and other types of anxiety disorders [36,37,38,39,40,41,42], depression [43,44,45,46,47,48,49,50,51,52], and stress [53,54].

The recognized advantages of using ICTs do not refer merely to their power to compensate for the limitations of traditional interventions (e.g., travelling requirements for customers or therapists), nor to their use as complementary means. There are several advantages associated with implementing internet interventions [8,9,10,11,13,14,55]. These include easy accessibility, high adaptability, flexibility and convenience, evolution at the client’s pace, easy adherence and treatment monitoring, privacy and possibility of anonymity, cultural adaptability, low cost, and high potential for dissemination [55]. Conversely, the main challenges identified in the use of ICTs include ethical concerns (e.g., security, privacy, confidentiality, and an absence or lack of deontological orientation), clients’ ICT illiteracy, and negative attitudes toward internet interventions [6]. Others can be added, such as a lack of access to technological and digital tools by some users, technological problems in their use, and changes to the setting and regarding the therapeutic relationship [8,10,14,26,55,56,57,58].

Because different circumstances and factors seem to contribute to explain psychologists’ attitudes and the adoption (or not) of this type of resources, it is important to identify potential changes in the use of ICTs during the COVID-19 pandemic. Given the measures of physical distance and isolation that most governments imposed with the state of emergency (e.g., [4]), how have psychologists dealt with the provision of counseling and therapy to their clients? The aims of this work were to (a) analyze how the attitudes of professionals in the field of psychology have changed in relation to the use of ICTs in the context of psychological monitoring during the lockdown; (b) assess whether the practice of psychological counseling and therapy includes greater use of ICTs during the lockdown period; (c) identify the factors that potentially have affected such changes; and (d) study the possible adoption of guidelines for at-distance psychological monitoring by psychologists who are using ICTs during the period of physical distance

## 2. Materials and Methods

### 2.1. Participants

The sample in this study comprised 108 psychologists who were registered in the OPP. Most were women (89, or 82.4%). The mean age was 37.20 years old (*SD* = 10.05; *Min* = 23, *Max* = 65), with 55 (50.9%) in the age group between 23 and 35 years, and 53 (49.1%) in the group between 36 and 65 years. The number of years of professional experience ranged between one or less and 33 (*M* = 11.52; *SD* = 8.60). The sample reflects national representation (including continental Portugal and islands), with 19 of the 20 Portuguese districts participating in the study. The most represented districts were Porto (*n* = 39; 36.8%), Lisbon (*n* = 21; 18.9%), Braga (*n* = 13; 12.3%), and Coimbra (*n* = 9; 8.5%). These cities comprehend the national OPP delegations with the most psychologists [59]. Most participants held master’s degrees (*n* = 69; 63.9%). Most were specialists in clinical psychology (*n* = 60; 55.6%), and 23 (21.3%) had one or more advanced specialties, including in psychotherapy (*n* = 13; 12.0%), among others.

Participants worked mainly with adults (*n* = 81; 75.0%), followed by adolescents (*n* = 52; 48.1%), children (*n* = 46; 42.6%), and the elderly (*n* = 24; 22.2%), in the areas of anxiety disorders (*n* = 91; 84.3%), mood disorders (*n* = 72; 66.7%), personality disorders (*n* = 37; 34.3%), neurocognitive disorders (*n* = 30; 27.8%), among others. For more information, see Table 1.

### 2.2. Instruments

A questionnaire was developed for this study and a pilot test was conducted to check its comprehension level and adequacy for the current purposes. The questionnaire included 30 questions divided into three sections: (i) socio-demographic data, with 9 questions; (ii) experience before the COVID-19 pandemic, with 7 questions; and (iii) current experience using ICTs in psychology sessions (namely web-based interventions during the lockdown period), with 14 questions. The questions were designed based on a previous questionnaire developed for the purpose of studying the use of ICTs in the provision of therapy and counseling [14], as well as on the advantages and challenges identified in the literature about the use of these technologies on psychological counseling.

Socio-demographic data included gender, age, education level, number of years of professional experience, district where the respondent was practicing, area of specialization, and targeted population in the respondent’s practice (including development stages and most frequent disorders). The remaining two sections of the questionnaire focused on information about the use of ICTs in the respondents’ clinical practice. Questions included which tools and devices were used, clients’ degree of satisfaction with the use of ICTs, advantages and difficulties identified by the professionals, impact on clients’ adherence and on therapeutic results, among others (cf. Appendix A).

### 2.3. Procedure

This study was approved by the local ethics committee (Approval No.: CE0003A), and the questionnaire was made available at the web-based survey platform LimeSurvey [60]. OPP sent this anonymous online self-report questionnaire to its members via e-mail and published it on its webpage. The questionnaire was also sent to the authors’ professional institutions via their mailing lists and was made available via professional social media, such as LinkedIn. This procedure ensured that all psychologists registered at OPP (a mandatory requirement for practicing psychology in Portugal) were invited to take part in this study. The e-mail containing the questionnaire included the study’s description and aims, followed by an informed consent form. If the person agreed to participate, a link gave them access to the questionnaire. The data were collected during April and May 2020, precisely at the peak of the Covid-19 pandemic, and during the lockdown period. Data were exported from LimeSurvey [60] into IBM SPSS Statistics 25 Commuter License [61] for analysis.

### 2.4. Statistical Analysis

Descriptive statistics (e.g., frequency distributions) were conducted for the sample characteristics (e.g., age, gender, educational and professional background)) and for the data pertaining both to the period before and during the COVID-19 pandemic. Data before the COVID-19 pandemic included aspects such as the use of digital tools in professional practice, professional experience with this type of tool, and adherence of clients to therapeutic activities based on digital technologies. During the COVID-19 lockdown, analyses considered the following aspects: maintenance of psychological support services, percentage of clients who have maintained the use of psychological counseling or therapy, frequency and duration of the therapeutic sessions, therapeutic adherence, therapeutic relationship, feedback from the clients, and results of the at-distance sessions.

Additionally, a thematic analysis for the open-type questions was performed on the open-ended questions. Two independent raters (A.G. and I.P.C.) have proceeded to the classification of the categories in each answer. Conflicts were solved by a third rater (A.R.D.).

To explore the correlations between professional experience, age and the selected outcome variables, point-biserial and Spearman correlations were performed. 

## 3. Results

### 3.1. ICTs in Psychological Counseling and Therapy before the COVID-19 Pandemic

#### 3.1.1. Use of ICTs in Psychological Counseling and Therapy

Regarding the use of digital technologies for providing at-distance psychological counseling and therapy before the COVID-19 pandemic, most (*n* = 63; 58.3%) had rarely or never used digital tools in clinical practice before the COVID-19 pandemic (Table 2).

The reasons pointed out by Portuguese psychologists for never or rarely (*n* = 63) having used digital technologies in psychological counseling and therapy were they considered it very impersonal (*n* = 28; 44.4%), inefficient (*n* = 18; 28.6%), ineffective (*n* = 10; 15.9%), not safe enough (*n* = 9; 14.3%) or ethical (*n* = 8; 12.7%), and for their lack of knowledge on how to apply these technologies in psychological counseling and therapy (*n* = 1; 1.6%). Under “Other”, participants additionally shared that they did not feel the need to use these means of providing counseling and therapy before, that these means were not part of their institutions’ policies, and that they preferred in-presence interventions.

#### 3.1.2. Tools and Technological Devices Used

Among the Portuguese psychologists who used ICTs to provide at-distance psychological counseling and therapy (*n* = 71), the most often used tools were video conferences (*n* = 50; 70.4%), telephone calls (*n* = 41; 57.7%), e-mails (*n* = 31; 43.7%), and social networks (*n* = 23; 32.4%). Other tools used were audio conferences (*n* = 14; 19.7%), online intervention platforms (*n* = 6; 8.5%), smartphones and tablet apps (*n* = 6; 8.5%), online forums (*n* = 4; 5.6%), chats (*n* = 3; 4.2%), short-message services (*n* = 2; 1.8%), and virtual rooms (*n* = 1; 0.9%). Concerning the technological devices used for providing at-distance psychological counseling and therapy, the most frequently used device was a computer (*n* = 62; 87.3%), followed by a telephone/smartphone (*n* = 58; 81.7%) and by tablets (*n* = 9; 12.7%) (Table 2).

#### 3.1.3. Psychologists’ Perceptions about Their Experiences and about Clients’ Adherence

Among the psychologists that used digital technologies in psychological counseling and therapy previously to the COVID-19 pandemic (*n* = 71), none considered their experiences with these tools to be negative or very negative. Nevertheless, 21 (of these 71) psychologists (29.6%) considered their experiences to be neither negative nor positive. Most of the respondents considered their experience with digital technologies to be either positive (*n* = 37; 52.1%) or very positive (*n* = 13; 18.3%). Regarding the involvement of their patients in the therapeutic activities that were delivered through digital technologies, most of the psychologists rated it as moderate (*n* = 28; 39.4%), followed by high (*n* = 18; 25.4%) and low involvement (*n* = 12; 16.9%). Only six psychologists (8.5%) considered their patients’ involvement in this type of activities very high, and seven psychologists (9.9%) rated their patients’ involvement as very low (Table 2).

#### 3.1.4. Advantages and Challenges of ICTs Use

With regard to the advantages that Portuguese psychologists considered might be experienced or already were experienced through the use of ICTs in psychological counseling and therapy, geographic flexibility was the most frequently selected advantage (*n* = 74; 68.5%), followed by scheduling flexibility (*n* = 55; 50.9%), the possibility of them reaching new groups of people in need of psychological counseling and therapy (*n* = 36; 33.3%), and by their easier access to some target-groups, such as persons with disability, refugees, among others (*n* = 28; 25.9%). They added the cost–benefit relationship (*n* = 27; 25.0%) and the possibility of obtaining new business areas (*n* = 10; 9.3%). Twelve (11.1%) of the respondents considered that they have never benefitted, or will never benefit, from any advantage through the use of ICTs in psychological counseling and therapy.

On the contrary, through the analysis of the challenges that psychologists had already faced or were afraid of facing when using new ICTs in psychological counseling and therapy, the most frequently referred challenge was the difficulty in establishing and/or maintaining the therapeutic relationship (*n* = 67; 62.0%), followed by the lack of non-verbal communication (*n* = 66; 61.1%), reduced therapeutic adherence (*n* = 52; 48.1%), reduced client engagement in the sessions (*n* = 50; 46.3%), and reduced privacy (*n* = 35; 32.4%). Other challenges referred by the psychologists were the interruption of the sessions (*n* = 29; 26.9%), ethical concerns (*n* = 23; 21.3%), possible misunderstandings (*n* = 22; 20.4%), difficulties in therapeutically approaching some problems/topics (*n* = 20; 18.5%), the substantial decrease or increase of the sessions’ frequency (*n* = 20; 18.5%), lack of security (*n* = 19; 17.6%), establishment of boundaries (*n* = 19; 17.6%), and time management (*n* = 12; 11.1%). Under the category “Other”, they also mentioned technical problems. Four (3.7%) respondents considered to never have faced or feared to face challenges in the future related to the use of digital technologies in their professional practice (Table 2).

### 3.2. ICTs in Psychological Counseling and Therapy during the COVID-19 Pandemic

#### 3.2.1. Use of ICTs in Psychological Counseling and Therapy

During the COVID-19 pandemic, and specifically during the lockdown period, only 17 (15.7%) of the 108 psychologists discontinued the provision of psychological counseling and therapy to their clients (Table 2). These psychologists reported that the main reasons for interrupting their professional activities were the suspension of activities on the part of the institution where they worked, activity suspension on the part of their clients for various reasons (e.g., considering themselves to be info-excluded populations or presenting digital illiteracy, financial difficulties, or sensing that the clinical setting is lacking), psychologists’ own personal unavailability during this period (e.g., due to new family responsibilities), and considering that digital means were inadequate for the target population (i.e., children) or clinical condition (e.g., attention deficits) that they were treating. All the other psychologists (*n* = 91, 84.3%) were able to continue the sessions with their cases due to the use of ICTs.

#### 3.2.2. Readiness for the Use of ICTs

Among the 91 psychologists who continued to provide at-distance psychological services, 71 (84.3%) previously read guidelines and other documents that support their at-distance psychological practice. The documents that these psychologists consulted the most were materials made available by the OPP (e.g., written material, videos, and webinars), guidelines from APA and from the International Psychoanalytical Association (IPA), scientific papers, and manuals about online therapeutic interventions (including the Therapy2.0 project).

Regarding additional cautionary procedures implemented by the psychologists for at-distance interventions, respondents referred the careful definition of rules and ethical limits, namely in terms of privacy, confidentiality, security, schedules, forms of contact, session duration and frequency, as well as how to proceed when unforeseen situations occur (e.g., technical failures such as problems with the internet connection, technology problems such as problems with the tools/equipment used, or interruptions). Caution about the type of software and the type of technology used were mentioned, also related with non-exposure of personal life, as well as the conditions of the physical space and the psychologist’s personal appearance/presentation, and personal well-being. Psychologists also referred several precautions and procedures associated with the actual therapeutic process, namely regarding verbal and non-verbal communication (e.g., minimizing the occurrence of overlaps, interruptions, and misunderstandings), greater session structuration and directivity (which involved greater previous preparation for some of them), avoidance of emotional themes that require in-person support, which distance prevents, parent follow-up in sessions with children, and assessment of clients’ level of comfort with the new format.

#### 3.2.3. Tools and Technological Devices

When focusing on the technological tools used to provide at-distance psychological counseling and therapy, video conference was the most frequently used (*n* = 71; 78.0%), followed by phone calls (*n* = 48; 52.7%), social networks (*n* = 35; 38.5%), e-mail (*n* = 33; 36.3%), audio conference (*n* = 16, 17.6%), smartphones and tablet apps (*n* = 7; 7.7%), online intervention platforms (*n* = 3; 3.3%), chats (*n* = 3; 3.3%), online forums (*n* = 1; 1.1%), and virtual rooms (*n* = 1; 1.1%). Computers were the most frequently used technological device to provide psychological services during the COVID-19 pandemic (*n* = 80; 87.9%), followed by telephones/smartphones (*n* = 66; 72.5%) and tablets (*n* = 9; 9.9%) (Table 2).

#### 3.2.4. Impact of the COVID-19 on the Psychologists’ Practice

Most of the respondents (*n* = 53; 58.2%) have continued to provide their services to most of their clients, i.e., twenty-seven (29.7%) of the psychologists continued to provide counseling and therapy to between 51% and 75% of their clients, and 26 psychologists (28.6%) to between 76% and 100% of their clients. However, for 23 psychologists (25.3%), the number of clients decreased to a range of between 0% and 25%, and for another 15 psychologists (16.5%) that number diminished to a range of between 26% and 50%. These psychologists referred, as main reasons for these reductions, low client adherence, lack of client’s necessary privacy, confidentiality and non-interruption conditions at home, the fact that clients preferred in-presence contacts (considering such forms of intervention to be more effective than, or feeling uncomfortable with, the new format), had financial difficulties, had difficulties managing the new routines (including caring for the children at home), and lacked the technological means for at-distance sessions. In some cases, the client’s condition was stable, and the therapeutic process had come to an end, or it was requiring no immediate sessions.

Considering the frequency of the counseling and therapy sessions among the clients who continued to use this service, a small majority of the psychologists (*n* = 48; 52.7%) referred that their clients have maintained the previous frequency, but 29 psychologists (31.9%) reported a decrease in the number of sessions, and six (6.6%) reported a significant decrease in that number. Despite that, seven psychologists (7.7%) reported an increase in the number of sessions during the COVID-19 pandemic, and one (1.1%) reported a significant increase. The same pattern was found for the duration of the counseling and therapy sessions, with 55 (60.4%) psychologists reporting a maintenance of the duration of each session, 20 (22.0%) reporting a decrease, and 4 (4.4%) reporting a significant decrease in the duration of the sessions. Nevertheless, 12 psychologists (13.2%) stated that the duration of the counseling and therapy sessions increased during the COVID-19 pandemic.

#### 3.2.5. Psychologists’ Perception about Their Experiences and about Clients’ Adherence

Regarding the results of the current therapeutic sessions, when compared to former in-presence sessions, most psychologists (*n* = 65; 71.6%) considered the results to be more of less the same, four (4.4%) reported obtaining better results with at-distance sessions, and 22 (24.2%) considered that at-distance sessions have yielded worse results than in-presence sessions. Similarly, from the points of views that clients shared with their psychologists, at-distance and in-person sessions were more or less the same (*n* = 71; 78.0%). Six (6.6%) of the respondents reported receiving better feedback (i.e., the clients preferred the online sessions), and one (1.1%) received much better feedback. Even so, 13 (14.3%) psychologists received worse feedback from their clients about this type of intervention.

In what concerns therapeutic adherence to the ICT sessions, the majority of psychologists considered it to be more or less the same during the COVID-19 pandemic, comparing to the pre-COVID sessions (*n* = 52; 57.1%), and 10 psychologists (11.0%) reported an improvement. Nevertheless, other psychologists reported a decrease (*n* = 24; 26.4%) or a significant decrease (*n* = 5; 5.5%) in the therapeutic adherence of their clients (Table 2). The vast majority of respondents considered that the therapeutic relationship between the psychologists and their clients was maintained (*n* = 77; 84.6%), with only three (3.3%) psychologists reporting an improvement in those relationships. However, 11 (12.1%) psychologists considered that those relationships have worsened during this period.

#### 3.2.6. Advantages and Challenges of the ICTs Use

Regarding the advantages that Portuguese psychologists viewed as associated with their current use of new ICTs in psychological counseling and therapy, geographic flexibility was the most frequently selected (*n* = 73; 80.2%), followed by scheduling flexibility (*n* = 57; 62.6%) and the possibility of reaching new groups of persons in need of psychological counseling and therapy (*n* = 30; 33.0%). Other advantages that they mentioned were the cost–benefit relationship (*n* = 24; 26.4%), the easier access of psychologists to some target groups, such as persons with disability and refugees, among others (*n* = 21; 23.1%), and the possibility of obtaining new business areas (*n* = 11; 12.1%). Under the category “Other”, they further mentioned the possibility of providing secure interventions in the current COVID-19 pandemic context, which ensured the possibility of maintaining the interventions. Only two (2.2%) respondents considered that at-distance psychological counseling and therapy does not offer any advantages.

Through the analysis of the challenges that psychologists currently face when they provide at-distance psychological counseling and therapy sessions, the most frequently referred difficulty was lack of non-verbal communication (*n* = 58; 63.7%), followed by reduced privacy (*n* = 36; 39.6%), the difficulty in establishing and/or maintaining the therapeutic relationship (*n* = 34; 37.4%), session interruptions (*n* = 31; 34.1%), reduced therapeutic adherence (*n* = 22; 24.2%), difficulties in approaching some problems/topics therapeutically (*n* = 19; 20.9%), ethical concerns (*n* = 19; 20.9%), and a reduction in patient engagement in the sessions (*n* = 18; 19.8%) (Table 2). Other challenges that counsellors referred to (under the category “Other”) were the establishment of boundaries (*n* = 17; 18.7%), the significant decrease or increase in session frequency (*n* = 16; 14.8%), the time management of the sessions (*n* = 15; 16.5%), possible misunderstandings (*n* = 14; 15.4%), and lack of security (*n* = 10; 11.0%). Under this category, they additionally mentioned technology problems (e.g., equipment adjustments) and technical failures (e.g., internet connection). Five respondents (5.5%) did not report any difficulty or challenge in providing at-distance psychological counseling and therapy.

In Table 2, the psychologists’ practices are presented pre- and post-COVID-19 for easy comparison of the main results described previously.

### 3.3. Variables Associated with Psychologist’ Attitudes and Practices

Significant point-biserial correlation coefficient were positive between the aspect “continue to provide psychological counseling to customers regularly” and years of professional experience, *r*_pb_ = 0.296, *p* = 0.002. Significant correlations were negative between “frequency of psychological counseling sessions” and both years of professional experience, *r*_s_ = −0.341, *p* < 0.001, and age, *r*_s_ = −0.229, *p* = 0.017. The aspect, “duration of psychological counseling sessions” also displayed a significant negative correlation with age, *r*_s_ = −0.209, *p* = 0.030. Thus, regarding respondents’ professional experience, psychologists with more years of experience maintained their professional services during the COVID-19 pandemic more than professionals with less years of experience. Nevertheless, the frequency of the sessions decreased for the professionals who had more years of professional experience. Regarding age, older psychologists reported a decrease in session frequency and duration. No significant results were found in any of the other variables analyzed.

## 4. Discussion

This study aimed to explore psychologists’ attitudes and practices related with the use of ICTs before and during the COVID-19 pandemic lockdown period, for identification of the main changes that have occurred in the provision of counseling and therapy. The impact of age and years of professional experience on the use of ICTs was also inspected.

In this study, psychologists’ use of ICTs in their professional activity before the COVID-19 pandemic is in accordance with the literature, namely in terms of previous experience of their use, tools, devices, professionals’ satisfaction with their use, advantages, and perceived challenges. These results reproduce those by Mendes-Santos (2020), in which only 29.6% of the inquired Portuguese psychologists admitted to having used digital technologies in their professional practice. The results of the present study also showed that most Portuguese psychologists had never or rarely used digital technologies as a means of delivering psychological counseling and therapy before the COVID-19 pandemic [6]. There is also a high degree of similarity between the tools most frequently used in our study and the tools used, the resources that psychologists most recommend to their clients (e.g., telephone calls, e-mails, video conferences, social networks, and apps) [6], and the most used devices (e.g., computers and smart phones) reported in other research (e.g., [14]). Additionally, according to previous studies, accessibility/geographic flexibility, convenience/(scheduling) flexibility, and cost-effectiveness/low cost are amongst the most recognized advantages of using ICTs (e.g., [6,55]). Regarding the disadvantages, the major challenges in this study, pertaining to ethical concerns and to the difficulty in establishing and/or maintaining the therapeutic relationship due to different reasons, were also identified in the literature [6,8,10,14,26,55,56,57,58].

The analysis of the reasons given for not using ICTs before the COVID-19 pandemic revealed that lack of knowledge and training about the correct use of ICTs was particularly relevant, which might explain professionals’ concerns about efficiency, effectiveness, and ethical issues. These same factors were associated with more negative attitudes toward the use of technologies in a previous study [6]. Training thus seems to be a necessary step in order to increase the use of ICTs. However, before the COVID-19 pandemic, available training was scarce, perhaps also because the professionals themselves perceived the use of ICTs as unnecessary, as they indicated in this study.

The emergence of the COVID-19 pandemic brought about relevant changes in the use of ICTs. Barriers to their use by both professionals and clients have been reduced, as the availability of information about their use in various formats has increased. The percentage of psychologists who have adopted ICTs in their practice during the lockdown period was very high in this study, and the vast majority of respondents were able to maintain their professional activity due to the inclusion of these means in their practice. This shows an enormous capacity of adaptation and flexibility, both on the psychologists’ and clients’ parts. This phenomenon was observed not only in Portugal but in other countries too, such as the United States, where the provision of at-distance psychological services has been raised from 7.07% to 85.53% [62]. Additionally, other health services are also adopting the online modalities, namely in medicine [63,64], with the professionals reporting positive perceptions regarding the telehealth services. However, the implementation of ICTs in such a short period of time leads to questions about the conditions under which they were implemented.

Our results showed that more than half of the psychologists have read about the use of ICTs, and some had already used these tools, even if not exclusively, in their professional practice before, which is consistent with a previous study [6]. They additionally identified a number of materials that were informative of at-distance psychological practice. These materials also contained information on additional care that needs to be adopted in at-distance psychological monitoring sessions and that is different from the procedures that these professionals might have adopted in the use of digital technologies in the context of their social relationships.

Their concerns about web-based session pertained to a diversity of aspects considered to be critical in the e-Health literature (e.g., the clear definition of rules and ethical limits) that can be different from face-to-face services [13]. However, it is noteworthy that most professionals have not offered any input on any additional measures that they might have adopted (e.g., use end-to-end encrypted technology), nor has it been possible, within the scope of this work, to identify how psychologists were capable of responding effectively to the new requirements and specificities that they reported they have adopted. Despite the great availability of webinars and the training and specialty documents made available during this period (e.g., [13]) by accredited entities, often free of charge, little is known about how such information transfer to the professional contexts.

This study provides important information in that regard by confirming the pertinence and usefulness of such materials among the psychologists. In general, the tools and devices used before the lockdown period were the same that were used during the COVID-19 pandemic, although there was an increase in the use of several of them during the COVID-19 pandemic (e.g., video conferences, computers, and telephones/smartphones). The primary use of computers and smartphones in this study is in line with the findings from previous research, although psychologists in our study used mostly video conference and telephone calls, whereas e-mail was the most widely used tool in a previous study [14].

Psychologists who do use ICTs in their practice tended to report a positive or very positive experience regarding the use of these online technologies in counseling and therapy. Similarly, a study focused on the attitudes of psychotherapists towards online therapy during the COVID-19 pandemic have also found a positive attitude of the professionals with regard to this therapy modality [65]. Research has recognized several advantages associated with using ICTs in such contexts, and participants in our sample identified equivalent advantages [8,9,10,11,13,14,55]. Their reported advantages were the same before and during the lockdown period, although they added a new advantage during the lockdown period, i.e., the possibility to conduct secure interventions. The number of professionals who did not see any advantage in the use of ICTs after the pandemic decreased to a practically residual value. However, the implementation of these modalities was not without difficulties. Both before and during the pandemic, psychologists identified a set of challenges in the adoption of DICTS in professional practice. Some decreased during the pandemic, possibly due to the professionals’ increased experience (e.g., establishing/maintaining the therapeutic adherence). However, others were particularly worsened during the period of mandatory lockdown (e.g., loss of privacy and risk of interruption).

Although generally positive, the results regarding psychologists’ experiences/results and clients’ adherence to therapeutic activities based on digital technologies were variable, as reported in previous studies (e.g., [36,37,38,39,40,41,42,43,44,45,46,47,48,49,50,51,52,53,54] and [6,55] respectively). It is important in the future to understand which ingredients explain this variability, both in relation to individual and to disorder aspects, and to work with the group of clients who have failed to adhere to the new format. From the limitations identified in this study, some clients might benefit from better advertisement of this type of services and of the scientific evidence of its effects, together with the possibility of receiving a reduction in the price of the services provided. Increasing clients’ digital literacy will also contribute to their adherence to web-based interventions. This is already happening among the new generations, whose members are already known as digital natives [14,15], but it is still difficult when working with specific populations, such as the elderly and people that live in rural areas, or when performing some psychological acts, such as psychological assessment and rehabilitation practices [62]. Additionally, other challenges might be more difficult to overcome, such as the sense that an adequate therapeutic setting is lacking. This aspect has been particularly exacerbated by the situation of mandatory lockdown that has brought together all who live in the same physical space, namely risking privacy.

Regarding the correlations between the professionals’ characteristics (i.e., age and years of professional experience) and the use of ICTs in professional practice, the results showed that were the professionals with more professional experience who presented greater maintenance of psychological support services, but less frequently. However, because it was also the professionals with more professional experience who presented greater maintenance of psychological support services, the decrease in the frequency of the sessions reported by them might have been an intentional procedure to help their own and their clients’ adaptation to the new format. 

The results failed to show a correlation between age and the use of ICTs, except for frequency and duration of the therapeutic sessions, which was significantly shorter for the oldest than for the youngest psychologists. The decrease in these aspects in the group of older psychologists from the pre- to the during-lockdown period could be possibly explained by the greater discomfort that these professionals experienced with the use of ICTs. The influence of the personal characteristics of the professionals in their attitude towards online psychological counseling was also reported in another study [65], with the psychotherapists who had previous experience with online psychotherapy, who thought that the patients they attend to had positive experiences in this modality, that adopted cognitive behavioral therapy in their practices (in comparison to psychodynamic therapists), and that lives in North America (in comparison to Europe) exhibiting a more positive attitude towards online psychotherapy.

This work has some limitations, namely the sample size and the use of a questionnaire that has not been previously validated to study the attitudes of psychologists toward ICTs (namely toward web-based interventions). However, the data collection took place during the period of absolutely unique and exceptional sanitary measures to prevent the pandemic dispersion of the coronavirus SARS-CoV-2, which causes COVID-19. To understand the impact of these circumstances on professionals’ practice is of the utmost relevance, despite the fact that these same circumstances have limited the time to conduct the data collection and the availability of participants in the study. Still, the collaboration of the OPP in this scenario, advertising the study and making the questionnaire available to all its members, contributed to ensure national representation of the participants. The process of adapting an existing instrument, namely obtaining the respective authorizations, would require an extended period of time that would risk missing this window of opportunity. Instead, the questionnaire was adapted from an instrument that was previously used by the authors and that was tested in a pilot-study. Future studies could focus on exploring the reasons that seem to be interfering either negatively or positively with clients’ adherence to, and satisfaction with, ICT sessions, so that personalized healthcare services can be provided and tailored to the specificities of each case.

## 5. Conclusions

It is widely known that the COVID-19 pandemic and associated restrictive measures of physical contact have significantly changed many professional activities. The current work has contributed to our understanding of that impact in the practice of psychology and psychotherapy, in close relation with the use of ICTs. Awareness of these changes can guide future professional practice by allowing the replication of the best practices and experiences shared by the psychologists during the period of maximum lockdown. It can also help to overcome the main difficulties and limitations experienced, for example, by guiding future training in this area, stimulating the creation of guidelines for ICT-based professional practice in different countries, and of measures to promote knowledge of and adherence to these guidelines that are becoming increasingly available.

## Figures and Tables

**Table 1:** Socio-demographic characteristics of the participants.

	*n*/%
	*M*/*SD*
Gender	M: *n* = 19, 17.6%
	F: *n* = 89, 82.4%
Age	*M* = 37.2; *SD* = 10.0
Education	Graduation: *n* = 26, 24.1%
	Master: *n* = 69, 63.9%
	PhD: *n* = 13, 12.0%
Professional Experience	*M* = 11.5; *SD* = 8.6
Clinical and Health Specialty	Y: *n* = 60, 55.6%
	N: *n* = 48, 44.4%
Advanced Specialties	Neuropsychology: *n* = 6, 5.6%
	Psychogerontology: *n* = 0
	Justice Psychology: *n* = 2, 1.9%
	Sports Psychology: *n* = 0
	Psychotherapy: *n* = 13, 12.0%
	Sexology: *n* = 1, 0.9%
	Others: *n* = 9, 8.3%
Population	Children: *n* = 46, 42.6%
	Adolescents: *n* = 52, 48.1%
	Adults: *n* = 81, 75.0%
	Elderly: *n* = 24, 22.2%
Psychological Disorders	Neurodevelopment disorders: *n* = 24, 22.2%
	Mood disorders: *n* = 72, 66.7%
	Anxiety disorders: *n* = 91, 84.3%
	Personality disorders: *n* = 37, 34.3%
	Substance-related disorders and addictive behaviors: *n* = 21, 19.4%
	Sleep disorders: *n* = 23, 21.3%
	Neurocognitive disorders: *n* = 30, 27.8%
	Others: *n* = 10, 9.3%

**Table 2:** Use of digital information and communication technologies in psychological counseling before and during the COVID-19 pandemic.

	Before the COVID-19	*n* (%)	During the COVID-19 Confinement Period	*n* (%)
ICTs use (*n* = 108)	never rarely sometimes frequently always	37 (34.3%) 26 (24.1%) 26 (24.1%) 14 (13.0%) 5 (4.6%)	yes (sessions continued) no (sessions discontinued during this period)	91 (84.3%) 15 (15.7%)
Tools (*n* = 71 before) (*n* = 91 after)	video conferences telephone calls e-mails social networks audio conferences online intervention platforms smartphones/tablet apps online forums chats short-message services virtual rooms	50 (70.4%) 41 (57.7%) 31 (43.7%) 23 (32.4%) 14 (19.7%) 6 (8.5%) 6 (8.5%) 4 (5.6%) 3 (4.2%) 2 (1.8%) 1 (0.9%)	video conference telephone calls social networks e-mails audio conference smartphones/tablet apps online intervention platforms chats online forums virtual rooms	71 (78.0%) 48 (52.7%) 35 (38.5%) 33 (36.3%) 16 (17.6%) 7 (7.7%) 3 (3.3%) 3 (3.3%) 1 (1.1%) 1 (1.1%)
Devices (*n* = 71 before) (*n* = 91 after)	computer telephone/smartphone tablets	62 (87.3%) 58 (81.7%) 9 (12.7%)	computer telephone/smartphone tablets	80 (87.9%) 66 (72.5%) 9 (9.9%)
Psychologists’ experiences/results (*n* = 71 before) (*n* = 91 after)	positive neither negative nor positive very positive very negative or negative	37 (52.1%) 21 (29.6%) 13 (18.3%) 0 (0%)	more or less the same better results worse results	65 (71.4%) 4 (4.4%) 22 (24.2%)
Clients’ involvement/adherence (*n* = 71 before) (*n* = 91 after)	moderate high low involvement very low very high	28 (39.4%) 18 (25.4%) 12 (16.9%) 7 (9.9%) 6 (8.5%)	more or less the same decreased improved significantly decreased significantly improved	52 (57.1%) 24 (26.4%) 10 (11.0%) 5 (5.5%) 0 (0.0%)
Advantages (*n* = 108)	geographic flexibility scheduling flexibility reaching new groups easier access to some target-groups cost-benefit relationship new business areas no advantages other	74 (68.5%) 55 (50.9%) 36 (33.3%) 28 (25.9%) 27 (25.0%) 10 (9.3%) 12 (11.1%)	geographic flexibility scheduling flexibility reaching new groups cost-benefit relationship easier access to some target-groups new business areas no advantages other	73 (80.2%) 57 (62.6%) 30 (33.0%) 24 (26.4%) 21 (23.1%) 11 (12.1%) 2 (2.2%)
Challenges (*n* = 108)	establishing/maintaining the therapeutic relationship non-verbal communication therapeutic adherence client engagement privacy session interruption ethical concerns misunderstandings difficulties approach some problems/topics sessions’ frequency lack of security establishment of boundaries time management no challenges other	67 (62.0%) 66 (61.1%) 52 (48.1%) 50 (46.3%) 35 (32.4%) 29 (26.9%) 23 (21.3%) 22 (20.4%) 20 (18.5%) 20 (18.5%) 19 (17.6%) 19 (17.6%) 12 (11.1%) 4 (3.7%)	non-verbal communication privacy establishing/maintaining the therapeutic relationship session interruptions therapeutic adherence some problems/topics ethical concerns patient engagement in the sessions other	58 (63.7%) 36 (39.6%) 34 (37.4%) 31 (34.1%) 22 (24.2%) 19 (20.9%) 19 (20.9%) 18 (19.8%)

## Appendix A

### The Use of New Digital Information and Communication Technologies in Psychological Counseling during the COVID-19 Pandemic

This project is conducted by researchers at the Center for Rehabilitation Research of the School of Health, Polytechnic Institute of Porto, and at the Laboratory of Neuropsychophysiology of the Faculty of Psychology and Education Sciences, University of Porto. Its main goal is to study the use of digital technologies in psychological counseling during the COVID-19 pandemic (SARS-CoV-2) in Portugal. To attain this goal, a questionnaire has been developed, targeting psychologists who are effective members of the Portuguese Psychologists Association.

Although the completion of the questionnaire is anonymous, some socio-demographic and professional data will be requested, as well as answers to closed- and open-ended questions. These questions focus on the use of digital technologies in psychological counseling. Please read each question carefully. It is important that your answers are sincere.

If you accept to participate in this study, please click on “Next” to proceed to the informed consent.

#### Informed Consent

All data will be collected and processed in an anonymous and confidential way, so you will never be asked for your name, professional number, or other personal data that could identify you. The data will be used for research purposes and will never be analysed at the individual level. Your participation is completely voluntary, and your contribution is very important for us, to better understand the role of digital technologies in confinement situations like the one we are going through now. Although you can quit answering the survey at any time without any consequences, we really appreciate your collaboration.

If you want to clarify any question or if you need more information, please contact: Andreia Geraldo (andreiageraldo.psic@gmail.com).

We appreciate your cooperation.

Andreia Geraldo, researcher

Artemisa R Dores, responsible for the project

If you intend to participate in this study, select the option below to proceed to the survey.

I declare that I have read the information above, have become aware of the research aims, and agree to participate in this study.

#### Socio-demographic and professional data

1. Gender:
  Man
  Woman
  Other
2. Age (years):
3. Education:
  Graduation
  Master’s
  PhD
4. Year of completion of the course [please consider the first degree that have allowed you to practice Psychology in Portugal (e.g., Pre-Bologna graduation; Post-Bologna Master’s Degree)]:
5. Number of years of professional experience [please count from the moment you started the practice of Psychology services]:
6. Distrit(s) in which you practice Psychology:
  Aveiro
  Beja
  Braga
  Bragança
  Castelo Branco
  Coimbra
  Évora
  Faro
  Guarda
  Leiria
  Lisboa
  Portalegre
  Porto
  Santarém
  Setúbal
  Viana do Castelo
  Vila Real
  Viseu
7. Are you a specialist in Clinical and Health Psychology accredited by the Portuguese Psychologists Association?
  Yes
  No
    (Note: In case the answer above is Yes, the next question appears)
  Do you have an advanced specialty recognized by the Portuguese Psychologists Association?
  Yes
  No
  If yes, which one(s) of them? (you can choose several)
  Neuropsychology
  Psychogerontology
  Justice Psychology
  Sports Psychology
  Psychotherapy
  Sexology
  Other
8. Select the age group(s) of the population your work with most often (you can choose several):
  Children
  Adolescents
  Adults
  Elderly
9. Indicate which psychological disorders you assess and intervene most often (you can choose several):
  Neurodevelopment disorders
  Mood disorders
  Anxiety disorders
  Personality disorders
  Substance-related disorders and addictive behaviours
  Sleep disorders
  Neurocognitive disorders
  Other(s): (please specify)
  Use of Digital Technologies in Psychological Counselling
  We ask you to consider your entire professional career as a psychologist, prior to the confinement measures imposed by the declaration of the State of Emergency in Portugal, to answer the following questions.
10. Have you used digital tools to conduct at-distance psychological counselling?
  Never
  Rarely
  Sometimes
  Often
  Always
  (Note: If the previous answer was Never or Rarely, the next question appears. If the answer is no, after responding to 10.1, the participants go directly to question no. 15)
  10.1. Which are the main reasons for you having never or rarely used this kind of tools?
    I don’t know how to apply them in my work
    I don’t consider them efficient (i.e., the adequate use of the necessary resources for the intervention/treatment)
    I don’t consider them effective (i.e., the ability of a given program to produce benefit when applied under ideal conditions)
    I find them very impersonal
    I don’t consider them safe enough
    I don’t consider them ethical enough
    I find them very expensive
    Other(s): (please specify)
11. Which tool(s) have you already used in at-distance psychological counselling?
  E-mail
  Audio-conference (e.g., Skype, Facetime, Zoom)
  Videoconference (e.g., Skype, Facetime, Zoom)
  Online platforms
  Online forum
  Chat
  Social networks (e.g., Facebook, Twitter, WhatsApp, Instagram, LinkedIn)
  Smartphone and tablet apps
  Virtual rooms (e.g., Second Life)
  Telephone calls
  Other(s): (please specify)
12. Which device(s) have you already used to maintain at-distance psychological counselling?
  Computer
  Tablet
  Telephone/smartphone
  Other(s): (please specify)
13. How do you rate your experience using this type of tools for conducting at-distance psychological counselling before the imposition of confinement measures by the declaration of the national state of emergency?
  Very negative
  Negative
  Neither negative nor positive
  Positive
  Very positive
14. How do you rate your clients’ involvement in therapeutic activities that use this kind of tools before the imposition of confinement measures by the declaration of the national state of emergency?
  Very low
  Low
  Moderate
  High
  Very high
15. What are the advantages that you have already benefited from, or that you consider you could have benefited from, by using this type of tools in psychological counselling, before the imposition of confinement measures by the declaration of the national state of emergency?
  Geographic flexibility, both for professionals and clients (i.e., they can interact from any location)
  Time flexibility
  Cost-benefit ratio
  To be able to reach new groups of people in need of psychological counselling
  Easy access to some target groups (e.g., people with anxiety, people with disabilities, refugees)
  New business areas
  None
  Other(s): (please specify)
16. Which are the challenges that you have faced or feared to face with the use of this kind of tools in psychological counselling processes before the imposition of the confinement measures by the declaration of the national state of emergency?
  Reduced therapeutic adherence
  Difficulty in establishing or maintaining the therapeutic relationship
  Significant decrease or increase in the frequency of the sessions
  Temporal management of the sessions
  Setting boundaries
  Interruption of the sessions
  Less client involvement in and commitment to the session
  Lack of non-verbal communication
  (Possible) misunderstandings
  Difficulty in therapeutically approaching a problem/topic
  Lack of security
  Reduced privacy
  Ethical concerns
  None
  Other(s): (please specify)
  Considering the challenges imposed to all psychologists by the COVID-19 pandemic situation (SARS-CoV-2), we ask you to answer the following questions according to your current professional practices.
17. Have you continued to provide psychological counselling to your clients regularly?
  Yes
  No
  (Note: If the answer to the previous question is no, the next question appears, and the participant has finished his/her questionnaire)
  17.1. Which are the reasons that have led you to suspend your professional activity?
18. Have you consulted any supporting documents or guidelines for at-distance psychological counselling or psychological counselling in crisis and catastrophe situations?
  Yes
  No
  (Note: If the answer to the previous question is yes, the next question appears)
  18.1. Please insert the name and/or link of the document(s) you consulted.
19. Currently, which percentage of your clients do you continue to monitor regularly when compared to the period before the declaration of the state of emergency in Portugal?
  Between 0% and 25%
  Between 26% and 50%
  Between 51% and 75%
  Between 76% and 100%
20. If there was a significant reduction in the percentage of clients that you regularly monitor, which are the main reasons that you identify for this to have happened?
21. The frequency of the psychological counselling sessions with each client that you continue to monitor
  Diminished a lot
  Diminished
  Remained the same
  Increased
  Increased a lot
22. The duration of the psychological counselling with each client that you continue to monitor
  Diminished a lot
  Diminished
  Remained the same
  Increased
  Increased a lot
23. Which tool(s) do you use to provide regular at-distance psychological counselling to your clients?
  E-mail
  Audio-conference (e.g., Skype, Facetime, Zoom)
  Video-conference (e.g., Skype, Facetime, Zoom)
  Online platforms
  Online forum
  Chat
  Social networks (e.g., Facebook, Twitter, WhatsApp, Instagram, LinkedIn)
  Smartphone and tablet apps
  Virtual rooms (e.g., Second Life)
  Telephone calls
  Other(s): (please specify)
24. Which device(s) have you already used to maintain at-distance psychological counselling?
  Computer
  Tablet
  Telephone/smartphone
  Other(s): (please specify)
25. How do you rate the therapeutic adherence of your clients currently, when compared to in-presence sessions?
  Much smaller
  Smaller
  More or less the same
  Greater
  Much greater
26. How do you rate the therapeutic relationship with your clients currently, when compared to in-presence sessions?
  Much worse
  Worse
  More or less the same
  Better
  Much better
27. How do you rate the results of each therapeutic session currently, when compared to in-presence sessions?
  Much worse
  Worse
  More or less the same
  Better
  Much better
28. How do you rate the feedback of your clients, when compared to in-presence sessions?
  Much worse
  Worse
  More or less the same
  Better
  Much better
29. In case you consider that you have adopted some additional precautions in at-distance psychological counselling, when compared to those you adopt in relation to the use of digital technologies in the context of your social relationships, please indicate them.
30. What are the advantages that you identify in at-distance psychological counselling?
  Geographic flexibility, both for professionals and clients (i.e., they can interact from any location)
  Time flexibility
  Cost-benefit ratio
  To be able to reach new groups of people in need of psychological counselling
  Easy access to some target groups (e.g., people with anxiety, people with disabilities, refugees)
  New business areas
  Other(s): (please specify)
31. Which are the difficulties that you face in promoting at-distance psychological counselling sessions?
  Reduced therapeutic adherence
  Difficulty in establishing or maintaining the therapeutic relationship
  Significant changes in the frequency of the sessions
  Temporal management of the sessions
  Setting boundaries
  Interruption of the sessions
  Less client involvement in and commitment to the session
  Lack of non-verbal communication
  (Possible) misunderstandings
  Difficulty in therapeutically approaching a problem/topic
  Lack of security
  Reduced privacy
  Ethical concerns
  Other(s): (please specify)
32. What are the difficulties that are reported by your clients in relation to maintaining at-distance psychological counselling?

Thank you for your collaboration.
